# P16 Centre of excellence on antimicrobial stewardship in Central Uganda

**DOI:** 10.1093/jacamr/dlad143.020

**Published:** 2024-01-03

**Authors:** David Musoke, Grace Biyinzika Lubega, Carol Esther Nabbanja, Suzan Nakalawa, Filimin Niyongabo, Jody Winter, Michael Brown Obeng, Claire Brandish, Kate Russell Hobbs, Ismail Kizito Musoke, Bush Herbert, Lawrence Mugisha, Linda Gibson

**Affiliations:** Makerere University, Uganda; Makerere University, Uganda; Makerere University, Uganda; Makerere University, Uganda; Makerere University, Uganda; Nottingham Trent University, UK; Nottingham Trent University, UK; Buckinghamshire Healthcare NHS Trust, UK; Buckinghamshire Healthcare NHS Trust, UK; Entebbe Regional Referral Hospital, Uganda; Makerere University, Uganda; Makerere University, Uganda; Nottingham Trent University, UK

## Abstract

**Background:**

Supported by a Commonwealth Partnership for Antimicrobial Stewardship (CwPAMS 2) grant, our partnership team comprising members from Makerere University, Nottingham Trent University, Buckinghamshire Healthcare NHS Trust and Entebbe Regional Referral Hospital (ERRH) seeks to scale up and enhance sustainability on antimicrobial stewardship (AMS) in Central Uganda. Building on the achievements of our previous CwPAMS projects, this current initiative aims at strengthening, human, animal and environmental health practitioners’ capacity on AMS in health facilities and the community, as well as promoting increased use of microbiology and prescribing data to inform clinical decisions.

**Objectives:**

To establish a centre of excellence on antimicrobial stewardship in Central Uganda using a One Health approach with a focus on capacity building, mentorship of lower-level facilities, and knowledge transfer.

**Methods:**

This project uses a One Health approach involving professionals from the domains of human, animal and environmental health. ERRH, the project hub, is currently mentoring lower health facilities (spokes) in AMS. AMS champions have been identified in each of these health facilities who are leading ongoing activities. The project has also employed CwPAMS AMS assessment tools during scoping visits to assess the baseline conditions at seven lower-level health facilities (one regional referral hospital, two district hospitals, three health centre IIIs and two health centre IIs). AMS workshops have so far been held in two districts (Nakaseke and Butambala) including two general hospitals.

**Results:**

The project held inception meetings that brought together different stakeholders in AMS. Scoping visits and AMS assessments have been successfully completed across seven health facilities, highlighting different AMS practices, challenges and intervention strategies. Furthermore, mentorship has so far resulted in establishment of AMS committees, identification of AMS champions, and adoption of the prescribing companion app at five of the seven mentored lower-level health facilities. AMS workshops held in the two districts resulted in increased knowledge on antimicrobial practices among the participants. The post assessments from the workshops showed that 73.4% of the participants had learnt the key importance of surveillance in AMS, 89.6% of the participants recognized the role of public awareness in promoting AMS, and 79.8% understood the value of infection prevention and control in promoting AMS in comparison with 51.7% at the pre-assessment (Figure 1). In addition, the online Community of Practice on AMS we established for health professionals in Uganda has seen a notable increase in membership, growing from 420 to over 600 members.

**Conclusions:**

By engaging directly with front-line health practitioners using a hub and spoke mentorship model, the early stages of this project have provided more information on the prevailing AMS challenges in Uganda, particularly knowledge and training gaps, while revealing promising opportunities such as the existing health care structures and supportive policies. AMS capabilities of health practitioners have been improved by participation in the project, but further work is needed to limit the development of AMR in Uganda.

**Figure 1.**
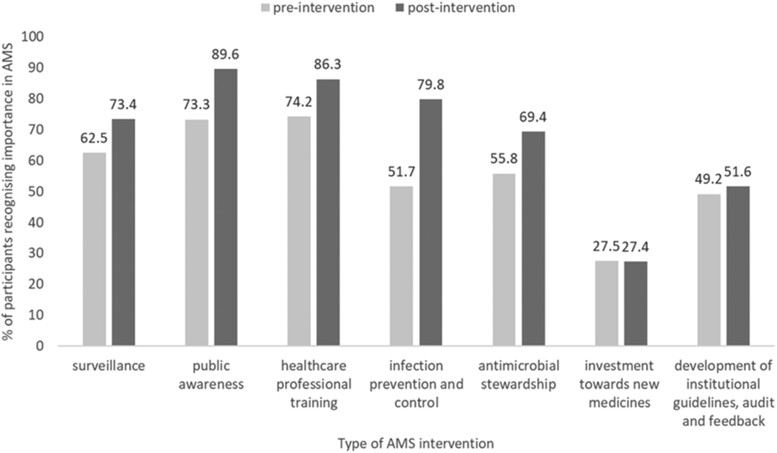
AMS indicators of health practitioners and community health workers who participated in training workshops demonstrating increased knowledge on antimicrobial stewardship practices after training.

